# Transcranial direct current stimulation and neuronal functional connectivity in MCI: role of individual factors associated to AD

**DOI:** 10.3389/fpsyt.2024.1428535

**Published:** 2024-08-19

**Authors:** Dong Woo Kang, Sheng-Min Wang, Yoo Hyun Um, Sunghwan Kim, TaeYeong Kim, Donghyeon Kim, Chang Uk Lee, Hyun Kook Lim

**Affiliations:** ^1^ Department of Psychiatry, Seoul St. Mary’s Hospital, College of Medicine, The Catholic University of Korea, Seoul, Republic of Korea; ^2^ Department of Psychiatry, Yeouido St. Mary’s Hospital, College of Medicine, The Catholic University of Korea, Seoul, Republic of Korea; ^3^ Department of Psychiatry, St. Vincent’s Hospital, College of Medicine, The Catholic University of Korea, Seoul, Republic of Korea; ^4^ Research Institute, NEUROPHET Inc., Seoul, Republic of Korea

**Keywords:** transcranial direct current stimulation, mild cognitive impairment, Alzheimer’s disease, resting-state functional connectivity, individual factors

## Abstract

**Background:**

Alzheimer’s disease (AD) encompasses a spectrum that may progress from mild cognitive impairment (MCI) to full dementia, characterized by amyloid-beta and tau accumulation. Transcranial direct current stimulation (tDCS) is being investigated as a therapeutic option, but its efficacy in relation to individual genetic and biological risk factors remains underexplored.

**Objective:**

To evaluate the effects of a two-week anodal tDCS regimen on the left dorsolateral prefrontal cortex, focusing on functional connectivity changes in neural networks in MCI patients resulting from various possible underlying disorders, considering individual factors associated to AD such as amyloid-beta deposition, *APOE* ϵ4 allele, BDNF Val66Met polymorphism, and sex.

**Methods:**

In a single-arm prospective study, 63 patients with MCI, including both amyloid-PET positive and negative cases, received 10 sessions of tDCS. We assessed intra- and inter-network functional connectivity (FC) using fMRI and analyzed interactions between tDCS effects and individual factors associated to AD.

**Results:**

tDCS significantly enhanced intra-network FC within the Salience Network (SN) and inter-network FC between the Central Executive Network and SN, predominantly in *APOE* ϵ4 carriers. We also observed significant sex*tDCS interactions that benefited inter-network FC among females. Furthermore, the effects of multiple modifiers, particularly the interaction of the BDNF Val66Met polymorphism and sex, were evident, as demonstrated by increased intra-network FC of the SN in female Met non-carriers. Lastly, the effects of tDCS on FC did not differ between the group of 26 MCI patients with cerebral amyloid-beta deposition detected by flutemetamol PET and the group of 37 MCI patients without cerebral amyloid-beta deposition.

**Conclusions:**

The study highlights the importance of precision medicine in tDCS applications for MCI, suggesting that individual genetic and biological profiles significantly influence therapeutic outcomes. Tailoring interventions based on these profiles may optimize treatment efficacy in early stages of AD.

## Introduction

Alzheimer’s disease (AD) involves the progressive accumulation of amyloid-beta (Aβ) peptide and phosphorylated tau proteins, which are pivotal in the cognitive decline observed in AD and its clinical precursor stages, including mild cognitive impairment (MCI) (1). MCI may evolve into dementia, affecting 10-15% of individuals annually (2). Despite some success in clinical trials of various drugs in slowing dementia progression, results are modest and highlight the need for more sustained and simpler interventions (3).

There is growing interest in monoclonal antibodies targeting Aβ for their potential to slow cognitive decline by 25-30% (4, 5), although their effects are less pronounced in MCI than in mild AD (6). This discrepancy highlights the need for comprehensive intervention strategies beyond pharmacological approaches, such as non-drug interventions that include cognitive activities, exercise, and dietary changes, which, despite their potential, present challenges in consistent application due to their complexity (7–9).

Among emerging therapeutic alternatives, non-invasive brain stimulation methods such as transcranial direct current stimulation (tDCS) show promise (10). Affordable and portable, tDCS can alter the excitability of cortical neurons (11), facilitate neuroplasticity (12), and potentially aid in the clearance of Aβ by affecting the blood-brain barrier (13). In the context of AD, changes in functional connectivity (FC), a critical indicator of the efficiency and integration of brain networks, reflect disease progression (14, 15). Therefore, maintaining or restoring FC is critical to mitigating the effects of AD (16). tDCS has shown potential in modulating functional brain network connectivity in patients with MCI, including those not pathologically confirmed to have AD but encompassing various potential underlying causes (17, 18). Specifically, high-resolution tDCS targeting the right parietal cortex significantly enhances segregation within the Default Mode Network (DMN) and the Dorsal Attention Network during spatial navigation tasks, suggesting a partial normalization of FC similar to patterns seen in cognitively intact individuals (19). While specific effects on the Central Executive Network (CEN) and Salience Network (SN) were less detailed, overall improvements in network segregation were observed, particularly during active task performance. This suggests that tDCS may be a promising therapeutic tool for restoring brain network functionality in early-stage neurodegenerative diseases.

In healthy older adults, tDCS has also been demonstrated to modulate both task-based and resting-state FC, leading to cognitive improvements. In the previous research, applying tDCS during working memory tasks has shown enhanced FC within the prefrontal cortex (20). Studies combining cognitive training with tDCS have reported increased FC between left and right brain regions within the CEN during tasks stimulating attention and processing speed (21, 22). Additionally, another study involving combined tDCS and cognitive training in older adults revealed increased resting-state FC within the CEN (23). These changes in both task-based and resting-state FC in healthy older adults have been associated with improvements in working memory. This context could provide a foundation for understanding the changes induced by a tDCS in MCI patients compared to healthy older adults.

Given the reported abnormalities in resting-state intra- and inter-network FC that correlate with MCI progression (15), examining changes after tDCS application may be clinically relevant. Despite the potential benefits, research in this area remains limited.

Precision medicine, which tailors therapeutic interventions based on individual genetics, biomarkers, and clinical profiles (24), could significantly improve the efficacy of tDCS. Factors such as Aβ deposition (25), presence of the *APOE* ϵ4 allele (26), brain-derived neurotrophic factor (BDNF) levels (27), and sex differences not only critically influence the progression of AD (28), but also influence treatment response. This necessitates the adaptation of tDCS protocols to individual factors associated to AD, potentially optimizing therapeutic outcomes in the clinical management of AD.

Our preliminary study in patients with MCI has provided important insights, showing that application of tDCS to the dorsolateral prefrontal cortex (DLPFC) results in variable effects on brain functionality (29). These differences are related to individual factors such as Aβ deposition and the presence of the *APOE* ϵ4 allele. Furthermore, studies suggest that the BDNF Val66Met polymorphism and sex differences may also significantly influence neuroplasticity and neurodegeneration after tDCS application, underscoring the heterogeneity of responses in MCI patients and the need for individualized treatment protocols.

In our ongoing study, we aimed to evaluate intra- and inter-network FC changes at rest in patients with MCI, including both positive and negative Aβ deposition groups, after 10 sessions of sequential anodal tDCS application to the left DLPFC, according to individual factors associated to AD such as Aβ deposition, APOE ϵ4 carrier status, BDNF polymorphism, and sex.

## Materials and methods

### Participants

The study enrolled subjects from the Brain Health Center at Yeoui-do St. Mary’s Hospital, which is affiliated with the College of Medicine of the Catholic University of Korea. Inclusion criteria included: 1) meeting Petersen’s MCI criteria (30), with inclusion of both amyloid-PET positive and negative cases, indicating a range of underlying conditions not exclusively due to AD and 2) having a Clinical Dementia Rating (CDR) of 0.5. Participants were excluded on the basis of: 1) a history of substance abuse, traumatic brain injury, or psychiatric illness; 2) current use of medications such as cholinesterase inhibitors, antidepressants, benzodiazepines, or antipsychotics; 3) contraindications to tDCS or MRI, such as having ferromagnetic or coiled metal implants; and 4) any dermatologic conditions affecting scalp skin integrity. The procedure was monitored by two experts in geriatric psychiatry. Participants consented to the use of their medical records for research purposes. All assessments took place at the same Brain Health Center. The study was conducted in accordance with the Declaration of Helsinki and was approved by the Institutional Review Board of the Catholic University of Korea (SC19DEST0012). Informed consent forms were signed by all study participants. The study was registered with the Clinical Research Information Service of the Korea Disease Control and Prevention Agency (KCT0006020) and was conducted from May 2020 to February 2022. There were no reported conflicts of interest related to the device manufacturers used in this study.

### Study protocol

In this single-arm, prospective study, we implemented a design that did not involve a sham condition. Participants received ten tDCS sessions in their homes, five times per week for two weeks. This regimen was selected based on previous clinical studies indicating that ten tDCS sessions were effective in treating symptoms of AD and MCI (31–34), while also taking into account compliance issues in the elderly. The decision to exclude a sham group was influenced by practical constraints, which precluded the possibility of administering sham-tDCS followed by real tDCS. Additionally, providing only sham-tDCS often led to low study enrollment among MCI patients, making it challenging to complete the project within the allocated timeframe.

The study did not utilize an “online” tDCS paradigm, where stimulation is applied during cognitive tasks, due to logistical challenges. Ensuring that all participants performed the tasks accurately and consistently during home sessions required additional staff and training resources, which were not feasible within our study design. Furthermore, the complexity of managing task performance concurrently with tDCS in a home setting presented significant difficulties in maintaining quality control and standardization.

**Figure 1:**
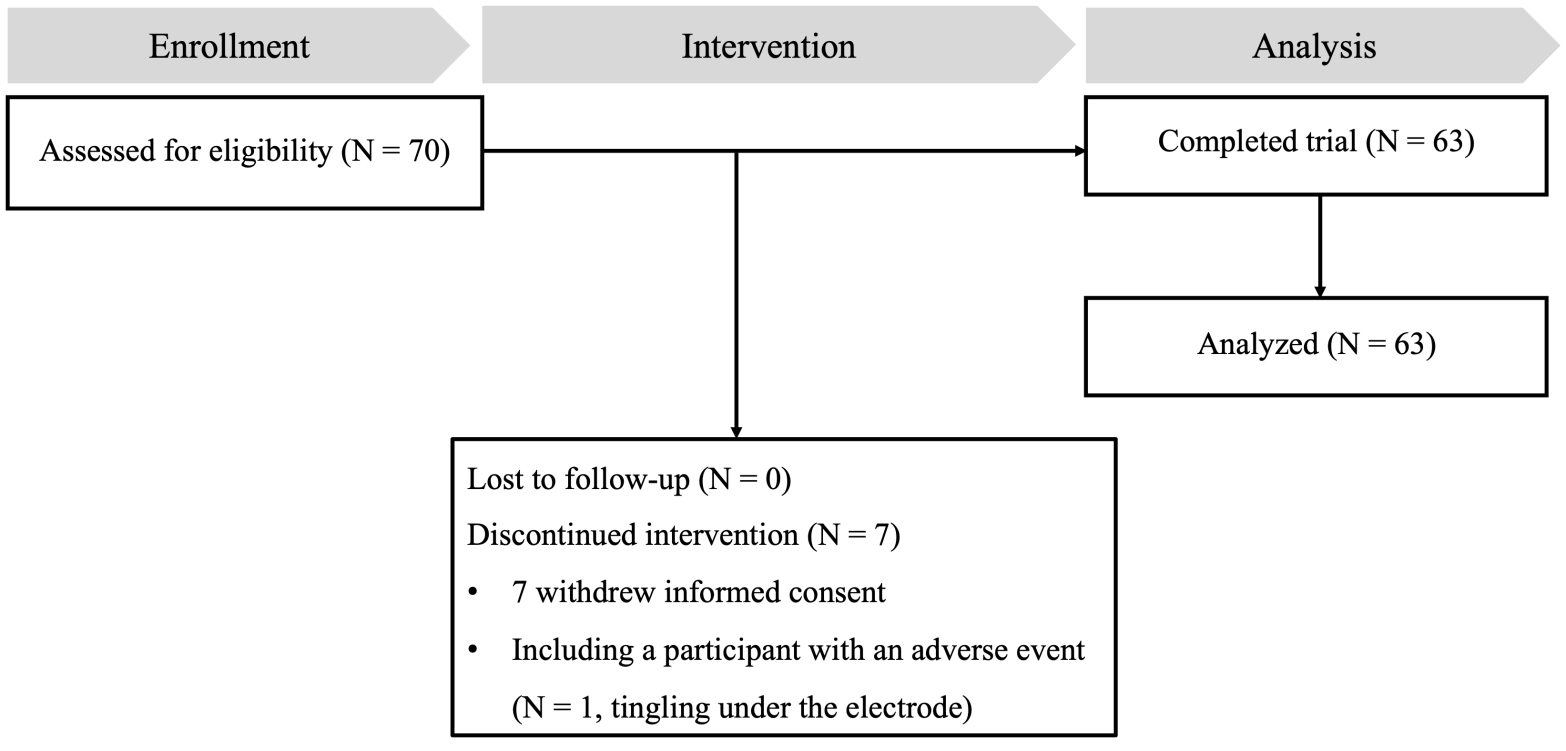
The flowchart of the study.

Neuropsychological assessments and MRI scans were performed at the Brain Health Center of Yeouido St. Mary’s Hospital, both within two weeks before the first tDCS session and after the tenth and final session. In addition, participants underwent [^18^F] flutemetamol (FMM) PET-CT scans and were tested for *APOE* and BDNF genes, all within four weeks before starting tDCS treatment. The results of the FMM-PET, *APOE*, and BDNF tests were not disclosed to the neuropsychological examiners or the participants. **Figure 1** shows a schematic diagram of the experimental protocols of the study.

### Transcranial direct current stimulation application

In this treatment protocol, a continuous direct current of 2 mA was delivered for 20 minutes using an MRI-compatible stimulator (YDS-301N, YBrain, Seoul, Republic of Korea). No in-scanner administration of tDCS was performed in this study. The anode was placed over the left dorsolateral prefrontal cortex (DLPFC), corresponding to the F3 location on the International 10/20 EEG system, and the cathode was placed over the right supraorbital area. The electrodes, which were disk-shaped with a radius of 3 cm, were applied to the scalp using saline-soaked sponge pads. Each session was conducted by trained personnel who visited the participants’ homes to ensure proper device operation. To ensure consistent electrode placement across all 10 sessions for each participant, staff used the anatomical landmarks of the International 10/20 EEG system, such as the nasion, inion, bilateral preauricular points, and vertex. The vertex is where the line from the nasion to the inion intersects the line connecting the preauricular points. The staff measured from a preauricular point to the center of the electrode, ensuring that it intersected with a line from the vertex to the nasion. They recorded the distance from both preauricular points to the center of the electrode and confirmed its relative position to these landmarks before each session. In addition, the accuracy of the electrode placement was checked 15 minutes after each session. The same staff member remained responsible for a given participant throughout all sessions.

### Neuropsychological assessment

All participants underwent cognitive assessments using the Korean adaptation of the Consortium to Establish a Registry for Alzheimer’s Disease (CERAD-K) (35). This included Korean versions of several tests: Verbal Fluency, the 15-item Boston Naming Test, and the Korean version of MMSE (MMSE-K) (36), as well as tests for word list memory, recall, recognition, constructional praxis, and recall. We also summed the scores of word-list memory, recall, and recognition to derive the total memory score. The total CERAD-K score was the aggregate of all scores, excluding the MMSE-K and CR. Cognitive function was evaluated using the Korean Montreal Cognitive Assessment (MoCA-K). For executive function, we utilized the Korean Stroop Word-Color Test (K-SWCT), which measures reaction control in letter and color reading scenarios (37), and Trail Making Test B, which measures the time taken to alternate letters and numbers in a sequence by drawing lines. Detailed descriptions of the assessments are provided in the **Supplementary Material**.

### *APOE* genotyping

The methodology for *APOE* genotyping is described in the **Supplementary Material**. Because of the protective effect associated with the *APOE* ϵ2 allele (38), subjects carrying this allele were excluded from the study. Participants were categorized according to the presence of the *APOE* ϵ4 allele; individuals with one or more ϵ4 alleles were classified as *APOE* ϵ4 carriers, whereas those without the ϵ4 allele were identified as non-carriers.

### BDNF genotyping

The procedure for BDNF genotyping is described in the **Supplementary Material**. With respect to the BDNF Val66Met polymorphism (rs6265), participants were classified into two groups based on recent genetic studies (39, 40): those with at least one Met66 allele were designated as Met carriers, and those without any Met66 alleles were designated as Met non-carriers.

### Magnetic resonance imaging acquisition

Imaging data were collected by the Department of Radiology of Yeouido Saint Mary’s Hospital at the Catholic University of Korea using a 3-T Siemens Skyra MRI machine and a 32-channel Siemens head coil (Siemens Medical Solutions, Erlangen, Germany). The scanning parameters of the T1-weighted three-dimensional magnetization-prepared rapid gradient echo sequences were as follows; echo time (TE)=2.6 ms, repetition time (TR)=1,940 ms, inversion time (TI)=979 ms, flip angle (FA)=9o, field of view=250×250 mm, matrix=256×256, and voxel size=1.0×1.0×1.0 mm^3^. Fluid attenuated inversion recovery (FLAIR) MRI sequences were as follows: TE= 135 ms; TR= 9000 ms; TI= 2,200 ms; FA=90o; FOV= 220x220 mm; matrix=356x231; and voxel size=1.0x 1.0x1.0 mm^3^. In addition, the scanning parameters of the DTI sequences were as follows; echo planar imaging, TR=3,100 ms, TE=86 ms, field of view=224 mm, voxel dimension=2 mm isotropic, B-value=1,000, gradients applied=64 isotropically, and distributed and acquisition time=5 min 44sec.

### Functional MRI data processing

Detailed methods for the acquisition of structural and functional MRI data are provided in the **Supplementary Material**. For the preprocessing of fMRI images, we used the Data Processing Assistant for rfMRI (DPARSF, GNU GENERAL PUBLIC LICENSE, Beijing, China), which operates within the framework of statistical parametric mapping (SPM 12, available at http://www.fil.ion.ucl.ac.uk/spm, Wellcome Centre for Human Neuroimaging, London, England). The preprocessing steps included slice timing adjustment, motion correction realignment, spatial registration, normalization, and smoothing. These procedures are thoroughly documented in our previous study and are also detailed in the **Supplementary Material**.

### Remote functional connectivity analysis: intra- and inter-network connectivity

We used three specific neural networks in our study - the DMN (41, 42), the CEN (43), and the SN (44)- that are known to be selectively impaired in AD. The 21 regions of interest (ROIs) for these networks were defined as 6 mm radius spheres centered on specific coordinates previously described in the literature (45). The coordinates for these ROIs, in Montreal Neurological Institute format, are listed in **Table 1**. For each of these resting-state networks, the within-network FC strength was determined by averaging the FC strength across ROIs within the same network ( $Z_{X}=\;\frac{1}{\frac{n_{X}\left(n_{X}-1\right)}{2}}\sum_{ij=1:n_{X}}\left|z_{i,j}\right|$ , where nX is the number of ROIs within a specific network X) (15). Similarly, the inter-network FC strength was calculated as the average of all possible connections between the ROIs in the different networks ( $Z_{X,Y}=\;\frac{1}{n_{X}n_{Y}}\sum_{i\in X,\;j\in Y}\left|z_{i,j}\right|$ , where X and Y denotes the network of the three resting state networks) (15).

**Table 1:** Location of Brain regions in resting-state brain networks.

ROI	MNI coordinates
Posterior cingulate cortex^a^	0, -51, 29
Medial prefrontal cortex^a^	0, 61, 22
Left lateral parietal^a^	-48, -66, 34
Right lateral parietal^a^	53, -61, 35
Left inferior temporal^a^	-65, -22, -9
Right inferior temporal^a^	61, -21, -12
Medial thalamus^a^	0, -9, 7
Left posterior cerebellum^a^	-28, -82, -32
Right posterior cerebellum^a^	26, -89, -34
Dorsal mPFC^b^	1, 30, 44
Left anterior PFC^b^	-45, 50, -5
Right anterior PFC^b^	46, 51, -7
Left superior parietal^b^	-51, -50, 49
Right superior Parietal^b^	53, -49, 47
Right anterior cingulate cortex^c^	12, 32, 30
Left anterior cingulate cortex^c^	-13, 34, 16
Right ventral anterior cingulate cortex^c^	10, 34, -6
Left putamen^c^	-19, 3, 9
Right putamen^c^	25, 18, 8
Left insula^c^	-42, 6, 4
Right insula^c^	43, 7, 2

^a^default mode network (DMN); ^b^central executive network (CEN); ^c^salience network (SN).

### [^18^F] flutemetamol PET image acquisition and processing

[^18^F] FMM was manufactured, and [^18^F] FMM-PET data were collected and analyzed as described previously (46). Static PET scans were acquired from 90 to 110 min after 185 MBq of FMM injection. MRI for each participant was used to co-register and define the ROIs and correct partial volume effects that arose from expansion of the cerebrospinal spaces accompanying cerebral atrophy using a geometric transfer matrix.

### SUVR calculation

The protocols for [^18^F] FMM PET imaging and the operation of the PET scanners are described in detail in the **Supplementary Material**. We quantified [^18^F] FMM uptake in PET/CT scans using standardized uptake value ratios (SUVRs), specifically targeting gray matter regions susceptible to Aβ plaques in early AD (46, 47). The pons, which is typically unaffected by Aβ deposition, was used as the reference region for calculating SUVRs. Regional SUVRs were calculated from the uptake ratios in cortical areas relative to the pons, and a composite SUVR was determined by averaging these values, taking into account the size of the regions involved. A threshold SUVR of 0.62 was used to discriminate between Aβ-positive and -negative accumulations, consistent with established benchmarks (46). In addition, PET scans were visually reviewed to ensure the accuracy of Aβ accumulation assessments.

### Statistical analysis

Statistical analyses were performed using R software (version 4.3.2) and jamovi (version 2.3.28) (https://www.jamovi.org). Normality assumptions were tested for continuous variables using the Kolmogorov-Smirnov test in the R software. All data had a normal distribution and were standardized for analysis using z-score transformation.

Repeated-measures ANOVA was used to predict the effect of the effect modifier by tDCS interaction (effect modifier × tDCS) on the z-transformed correlation values within and between the DEN, CEN, and SN neural networks, with tDCS (pre- and post-tDCS) as a repeated-measures factor and Aβ deposition, APOE ϵ4 carrier status, BDNF Val66Met polymorphism status, and sex as between-subject factors (potential effect modifiers). In addition, age, years of education, and other non-included effect modifiers were included as covariates. For the analysis of within-subject effects, if more than one effect modifier showed an interaction with tDCS application, we included the multiple effect modifier × tDCS interaction term (effect modifier 1 × effect modifier 2 × tDCS) in the analysis.

Significant interactions between effect modifiers and tDCS were observed in the z-transformed correlation scores within and between the DEN, CEN, and SN neural networks. For these neural networks of interest, further analyses were conducted to examine the impact of these interactions (differences in intra- and inter-network FC × effect modifier) on differences in neuropsychological performance. This subsequent analysis was performed using multiple regression, adjusting for variables such as age, years of education, and other non-included effect modifiers. All statistical analyses used a two-tailed P value< 0.05 to define statistical significance.

## Results

### Baseline demographic and clinical data

In this study, from the initial pool of 70 eligible participants, seven did not complete the study. Six participants voluntarily withdrew from the study, while one participant reported a minor side effect related to transcranial direct current stimulation (tDCS), experiencing a tingling sensation under the electrode. As a result, 63 participants successfully completed the study and were included in the final data analysis. **Figure 1** provides a comprehensive illustration of the participant flow throughout the study. Baseline demographic information for participants who completed the study is presented in **Table 2**.

**Table 2:** Baseline demographic and clinical characteristics of the study participants.

Demographic and clinical characteristics (N=63)	
Age (years)	73.2 ± 7.9
Gender	
- Male	21 (33.3%)
- Female	42 (66.7%)
Years of education	12.0 ± 5.0
[^18^F] Flutemetamol deposition (positivity, %)	26 (41.3%)
Global [^18^F] Flutemetamol SUVR_PONS_	0.62 ± 0.15
*APOE* ϵ4 carrier status (carrier. %)	30 (47.6%)
BDNF polymorphism (Val/Met or Met/Met, %)	52 (82.5%)
CERAD-K	
VF	12.1 ± 5.1
BNT	10.7 ± 3.1
MMSE	23.2 ± 5.0
WLM	14.6 ± 4.6
CP	10.1 ± 1.5
WLR	3.7 ± 2.6
WLRc	6.8 ± 2.8
CR	4.5 ± 3.4
TMT B (seconds)	224.1 ± 77.7
Stroop word-color	26.0 ± 14.2
Total memory	25.2 ± 8.8
Total CERAD-K	58.1 ± 15.2

Data are presented as mean ± SD unless indicated otherwise. SUVR_PONS_, standardized uptake value ratio of [^18^F] flutemetamol, using the pons as a reference region; CERAD-K, Korean version of the Consortium to Establish a Registry for Alzheimer’s Disease; VF, verbal fluency; BNT, Boston Naming Test; MMSE, Korean version of the Mini-Mental Status Examination; WLM, Word List Memory; CP, Constructional Praxis; WLR, Word List Recall; WLRc, Word List Recognition; CR, constructional recall; TMT B, Trail Making Test B; Total memory, composite score summing scores of the WLM, WLR, and WLRc tests; Total CERAD-K, composite score summing scores of the CERAD-K VF, BNT, WLM, CP, WLR, and WLRc domains.

### Changes in neuronal functional connectivity according to effect modifiers

**Figure 2:**
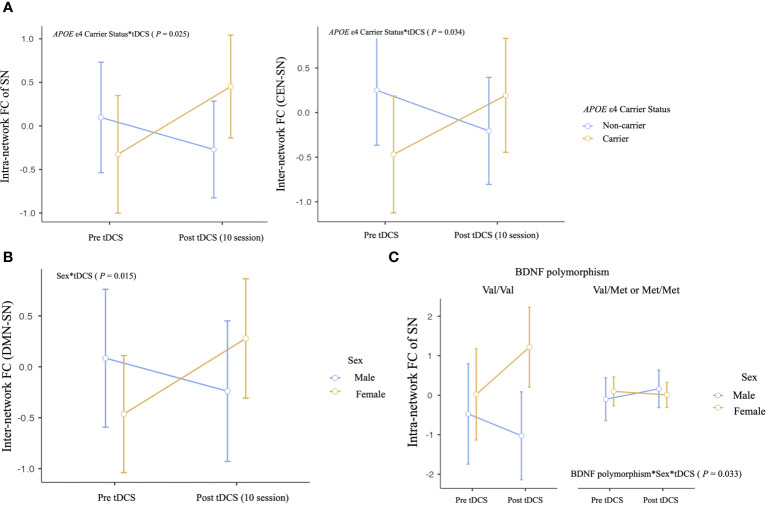
Differential impact of anodal tDCS on neural network functional connectivity according to effect modifiers: **(A)** *APOE* ϵ4 carrier status, **(B)** sex, and **(C)** multiple effect modifiers. Repeated‐measures ANOVA was used to predict the impact of effect modifier-by-tDCS interaction (effect modifier*tDCS) for neural network functional connectivity (Between-subject factors: Aβ deposition, *APOE* ϵ4 carrier status, BDNF polymorphism, and sex), adjusting for age, education years, and between-subject factors not showing interaction. DMN, default mode network; CEN, central executive network; SN, salience network.

We observed several important interactions in our study. First, a significant interaction was found between *APOE* ϵ4 carrier status and tDCS, as manifested by increased intra-network SN FC and inter-network CEN-SN FC of *APOE* ϵ4 carriers (intra-network SN FC, P = .025; inter-network CEN-SN, P = .034, **Table 3A**, **Figure 2A**). This indicates that following tDCS, a significant increase of intra-network SN FC and inter-network CEN-SN FC was observed in *APOE* ϵ4 carriers compared to *APOE* ϵ4 non-carriers. Additionally, a sex*tDCS interaction was identified, as evidenced by an increased inter-network FC of DMN-SN in female participants (P = .015, **Table 3B**, **Figure 2B**), suggesting that females demonstrated a significant increase in inter-network DMN-SN FC compared to males.

Table 3Differential impact of tDCS on changes in neuronal functional connectivity according to effect modifiers: (A) *APOE* ϵ4 carrier status, (B) Sex, and (C) Multiple effect modifiers.

**Table:** (A, C) Dependent variable: intra-network FC of SN.

	Sum of Squares	df	Mean Square	F	p
tDCS	0.35224	1	0.35224	0.6441	0.426
tDCS ∗ age	0.10457	1	0.10457	0.1912	0.664
tDCS ∗ education years	0.07565	1	0.07565	0.1383	0.711
tDCS ∗ Aβ deposition	0.00703	1	0.00703	0.0129	0.910
tDCS ∗ sex	1.15928	1	1.15928	2.1197	0.151
**tDCS ∗ *APOE* ϵ4 carrier status**	**2.91550**	**1**	**2.91550**	**5.3308**	**0.025**
tDCS ∗ BDNF polymorphism	0.10530	1	0.10530	0.1925	0.663
tDCS ∗ sex ∗ *APOE* ϵ4 carrier status	0.96004	1	0.96004	1.7554	0.191
**tDCS ∗ sex ∗ BDNF polymorphism**	**2.60993**	**1**	**2.60993**	**4.7721**	**0.033**
tDCS ∗ *APOE* ϵ4 carrier status ∗ BDNF polymorphism	0.87512	1	0.87512	1.6001	0.212

Type 3 Sums of Squares. Values in bold indicate statistical significance.

**Table:** (A) Dependent variable: inter-network FC between CEN and SN.

	Sum of Squares	df	Mean Square	F	p
tDCS	0.0598	1	0.0598	0.1053	0.747
tDCS ∗ age	0.9914	1	0.9914	1.7447	0.192
tDCS ∗ education years	0.2775	1	0.2775	0.4884	0.488
tDCS ∗ Aβ deposition	0.2598	1	0.2598	0.4572	0.502
tDCS ∗ sex	1.7437	1	1.7437	3.0685	0.086
**tDCS ∗ *APOE* ϵ4 carrier status**	**2.6936**	**1**	**2.6936**	**4.7402**	**0.034**
tDCS ∗ BDNF polymorphism	0.0331	1	0.0331	0.0582	0.810
tDCS ∗ sex ∗ *APOE* ϵ4 carrier status	0.4833	1	0.4833	0.8505	0.361
tDCS ∗ sex ∗ BDNF polymorphism	1.1389	1	1.1389	2.0042	0.163
tDCS ∗ *APOE* ϵ4 carrier status ∗ BDNF polymorphism	1.4830	1	1.4830	2.6098	0.112

Type 3 Sums of Squares. Values in bold indicate statistical significance.

**Table:** (B) Dependent variable: inter-network FC between DMN and SN.

	Sum of Squares	df	Mean Square	F	p
tDCS	0.45297	1	0.45297	1.07030	0.306
tDCS ∗ age	0.38077	1	0.38077	0.89970	0.347
tDCS ∗ education years	0.36850	1	0.36850	0.87070	0.355
tDCS ∗ Aβ deposition	0.00291	1	0.00291	0.00687	0.934
**tDCS ∗ sex**	**2.70389**	**1**	**2.70389**	**6.38881**	**0.015**
tDCS ∗ *APOE* ϵ4 carrier status	1.12444	1	1.12444	2.65687	0.109
tDCS ∗ BDNF polymorphism	1.17841	1	1.17841	2.78438	0.101
tDCS ∗ sex ∗ *APOE* ϵ4 carrier status	0.80497	1	0.80497	1.90201	0.174
tDCS ∗ sex ∗ BDNF polymorphism	1.06382	1	1.06382	2.51362	0.119
tDCS ∗ *APOE* ϵ4 carrier status ∗ BDNF polymorphism	1.04605	1	1.04605	2.47164	0.122

Type 3 Sums of Squares. Values in bold indicate statistical significance.

Repeated‐measures ANOVA was used to predict the impact of effect modifier-by-tDCS interaction (effect modifier*tDCS) for neural network functional connectivity (Between-subject factors: Aβ deposition, APOE ϵ4 carrier status, BDNF polymorphism, and sex), adjusting for age, education years, and between-subject factors not showing interaction. DMN, default mode network; CEN, central executive network; SN, salience network.

The tDCS* effect modifier variable is bolded if the p-value is less than 0.05.

Regarding multiple effect modifiers, our results showed a BDNF polymorphism*sex*tDCS interaction, which was seen as an increased intra-network FC of SN in female Met non-carriers (P = .033, **Table 3C**, **Figure 2C**). This finding indicates that female Met non-carriers experienced a significant increase in intra-network SN FC following tDCS compared to other groups.

Despite these significant findings, the effects of tDCS on FC did not differ between the group of 26 MCI patients with cerebral Aβ deposition detected by FMM PET and the group of 37 MCI patients without cerebral Aβ deposition. A table with all statistical comparisons shown in **Figure 2** is provided in the **Supplementary Results**.

### Association between changes in neuronal functional connectivity and neuropsychological performance scores: the role of effect modifiers

We identified significant interactions between tDCS and several effect modifiers, including *APOE* ϵ4 carrier status, sex, and BDNF polymorphism, in relation to changes in intra-network SN FC, inter-network CEN-SN FC, and inter-network DMN-SN FC. To determine whether these interactions also influenced neuropsychological performance scores, we analyzed the associations between changes in FC and performance scores with the same effect modifiers.

**Figure 3:**
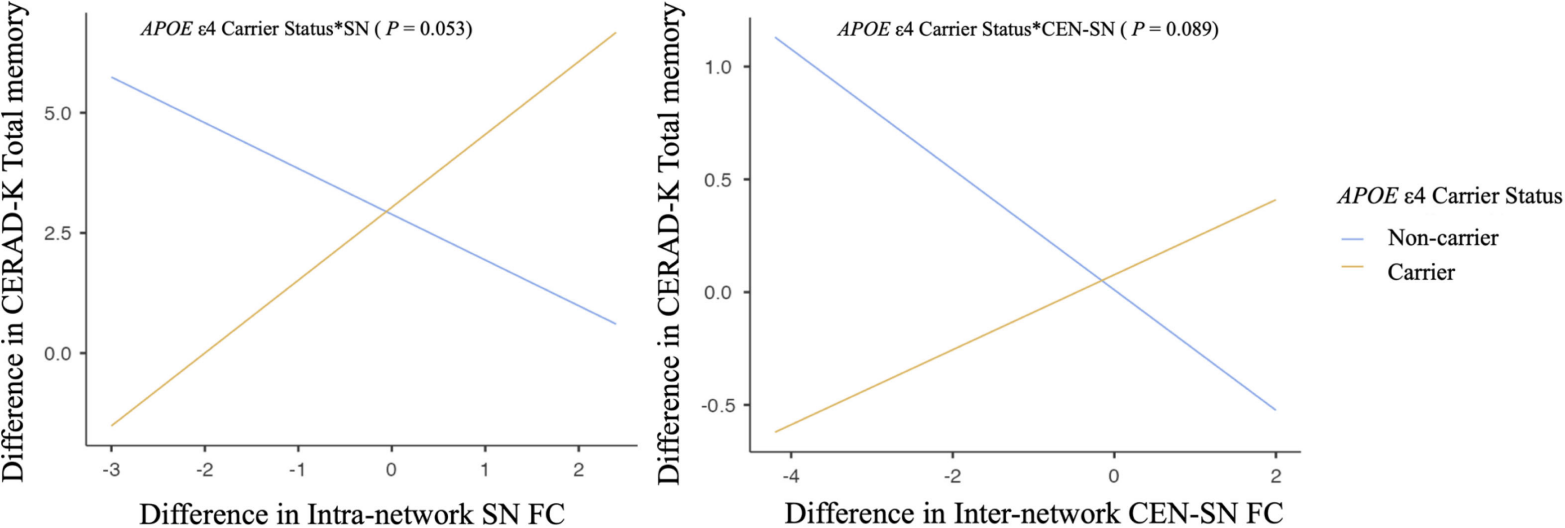
Association between changes in neuronal functional connectivity and neuropsychological performance scores according to *APOE* ϵ4 carrier status. Multiple regression analysis adjusting for age, years of education, and other nonincluded effect modifiers. SN, salience network; CEN, central executive network.

None of these interactions reached statistical significance for neuropsychological performance scores. However, some results indicated statistical trends (P values ranging from 0.05 to 0.1). Specifically, the interactions between changes in intra-network SN FC and inter-network CEN-SN FC with *APOE* ϵ4 carrier status showed trends toward significance in relation to CERAD-K total memory score (intra-network SN FC, P = .053; inter-network CEN-SN, P = .089, **Figure 3**). These trends suggest that *APOE* ϵ4 carriers exhibited a trend toward increased intra-network SN FC and inter-network CEN-SN FC, which was associated with improvements in CERAD-K total memory scores after tDCS compared with *APOE* ϵ4 non-carriers.

## Discussion

The aim of the current study was to evaluate whether a 2-week application of sequential tDCS alters intra- and inter-network FC changes at rest in patients with MCI and whether these changes depend on individual factors associated to AD. Furthermore, for network FCs that showed significant interactions with AD risk factors, we assessed whether the association with cognitive function scores was similarly dependent on AD risk factors.

In this study, we identified significant interactions between individual factors associated to AD and tDCS on resting-state FC in networks vulnerable to AD. Specifically, these interactions were observed as increased intra-network FC in the SN and inter-network FC between the CEN and SN in *APOE* ϵ4 carriers following sequential tDCS sessions compared with *APOE* ϵ4 non-carriers. This finding suggests a potential modulatory effect of tDCS on neural connectivity in *APOE* ϵ4 carriers.

The DMN, CEN, and SN are known to be well organized in cognitively normal status, but are reported to be disrupted as AD progresses (15). The SN, in particular, plays a central role in detecting and responding to salient stimuli, which is critical for efficiently directing cognitive resources (48). In the context of *APOE* ϵ4 carriers, increased SN connectivity may serve as an early marker of reorganization of the neural system in response to the initial pathological changes associated with AD (49). Such reorganization may be critical for maintaining cognitive function despite ongoing neurodegeneration (50). Therefore, the application of sequential anodal tDCS may have enhanced these neural adaptations in *APOE* ϵ4 carriers. SN is also central to switching between CON and DMN, facilitating cognitive control (48, 51). As AD progresses or as individuals approach the clinical thresholds of MCI and AD, significant disruptions in connectivity between key networks such as the SN and CEN become increasingly apparent (15, 45). Our results suggest that tDCS in MCI patients who are also *APOE* ϵ4 carriers, and thus at increased risk for AD, may strengthen SN-CEN inter-network FC, potentially aiding in the preservation of cognitive function. Although this study did not reach statistical significance, the observed positive correlation trends between total memory scores and both SN intra-network FC and SN-CEN inter-network FC in *APOE* ϵ4 carriers support our hypothesis. Further research with larger sample sizes and longer anodal tDCS durations may provide more definitive findings and elucidate the potential benefits of this intervention in *APOE* ϵ4 carriers of MCI patients.

In the present study, females demonstrated a significant increase in inter-network DMN-SN FC compared to males after sequential tDCS application, which was attributed to the sex*tDCS interaction. Connectivity between the DMN and SN is critical for cognitive function, as it integrates key networks involved in internal mental states and the detection of relevant external stimuli, thereby influencing cognitive processes such as attention, memory retrieval, and decision making (52–54). Enhanced DMN-SN connectivity is associated with improved cognitive flexibility and attention, which are critical for complex cognitive tasks and memory processes (55, 56). Previous research on sex differences in DMN-SN connectivity suggests that men often show more frequent switching between these networks and higher inter-network FC than women (57, 58). However, studies have shown that the SN, which is closely related to interoception, is more pronounced in females (59). These findings are mainly derived from studies conducted on younger adults and therefore need to be interpreted with caution when applied to older populations. In this context, the results of our study showing a significant increase in DMN-SN inter-network FC in females after anodal tDCS may imply a greater potential for improving DMN-SN inter-network FC in females compared to males. In addition, older women receive a higher intensity of tDCS current at the target site than men, possibly due to age-related sex differences in tDCS current intensity resulting from cerebral atrophy (60). This differential response may also contribute to the more pronounced increase in DMN-SN internetwork FC observed in women. Although this study found that DMN-SN inter-network FC varies significantly by sex under the influence of tDCS, it did not establish a direct link to differential cognitive function outcomes dependent on sex. This opens the door for further research to explore the potential cognitive effects of prolonged and intensified tDCS application in larger samples, which may help to clarify these initial findings.

This study also found that tDCS interacts with multiple factors to affect the intra-network FC of SN who were Val66 homozygotes and female following sequential anodal tDCS. This result suggests that female Met non-carriers showed a notable increase in intra-network SN FC after tDCS compared to the other groups. Regarding the sex* BDNF Val66Met polymorphism status, although some studies suggest a sexually dimorphic effect of the BDNF Met66 allele on AD susceptibility (61), others contradict this (62). Recent research using event-related functional MRI in young adults has shown that when presented with salient stimuli, male BDNF Met allele carriers show increased activation in the amygdala area, leading to successful encoding (63). The authors interpret these results as differences in BDNF activity-dependent secretion influenced by the Val66Met substitution, which may affect males and females differently due to different neural mechanisms or hormonal interactions. Although this functional MRI study shows a different pattern of interaction compared to the current study, it is noteworthy for demonstrating sex*BDNF Val66Met polymorphism status effects within regions belonging to the SN (64). The current study, which included an older cohort with MCI, may show different interaction characteristics than previous studies due to pathological aging. Given the age-related decline in BDNF levels, which is more pronounced in women (65), the application of tDCS, known for its potential to increase BDNF (66), may induce more pronounced neuroplastic changes in older women who are BDNF Val66Met homozygotes. In addition, our previous study analyzing changes in white matter microstructure showed similar patterns of sex*BDNF Val66Met polymorphism status in pathways critically involved in memory function and susceptible to AD (67). This suggests that variations in resting-state functional networks may align with microstructural changes in areas prone to neurodegeneration.

In our investigation, we observed no significant interaction between Aβ deposition and the effects of tDCS on resting-state intra- or inter-network FC. This intriguing result suggests that the neuromodulatory impacts of tDCS might not be substantially influenced by the presence of Aβ deposits. Notably, the association between Aβ deposition and resting-state FC was less consistent in MCI compared to dementia, with variations in FC also evident within MCI (68). Specifically, during the early phases of MCI, the brain may utilize compensatory mechanisms to maintain cognitive function, potentially masking or modulating the detrimental effects typically associated with Aβ accumulations (69, 70). Additionally, the impact of tauopathy, another core pathology of AD, on FC warrants consideration. While results have been somewhat mixed, numerous studies have reported that tau deposition contributes to increased FC (68). Given that the intensity of tauopathy can vary during the MCI phase, even in the presence of positive Aβ deposition (71), these factors may contribute to the observed lack of interaction between Aβ deposition and the application of tDCS. Therefore, evaluating the therapeutic effects of tDCS in MCI patients with tau deposition is essential to fully understand the influence of AD’s core pathologies on the therapeutic efficacy of tDCS.

Lastly, although there are differences in study design, studies in cognitively normal older adults have mainly reported changes in FC related to the CEN, often associated with improvements in working memory (20–23). However, in this study, the changes in FC varied depending on individual factors associated with AD. Increases in the SN were observed in *APOE* ϵ4 carriers, females, and BDNF Val66Met Met non-carrier females following tDCS. Additionally, although it is not directly related to changes in FC, lower baseline cognitive function has been reported to be associated with improved cognition following tDCS combined with a co-intervention. Therefore, the changes in FC in MCI patients, who have lower baseline cognitive function compared to cognitively normal older adults, may exhibit different patterns. To address these discrepancies, future studies should include larger sample sizes that encompass both cognitively normal older adults and MCI patients to explore these differences further.

A limitation of this investigation is the short duration of the anodal tDCS protocol. Our observations over a two-week tDCS regimen are consistent with previous research suggesting that duration influences the effect of memory enhancement (72, 73). Longer tDCS durations may reveal greater differences in brain functional changes associated with individual factors associated to AD. Future research should extend the duration of tDCS exposure and increase the number of participants to more fully explore these effects. Additionally, our study did not include information on the onset of cognitive decline, which is crucial for understanding the baseline differences among participants and their progression. Incorporating this information in future research would provide a clearer context for interpreting the effects of tDCS on brain functional changes associated with individual factors related to AD. In addition, the lack of a sham stimulation group was a deliberate choice focused on evaluating the moderating effects of individual factors associated to AD on the brain functional outcomes of tDCS, rather than a general oversight. Nevertheless, the inclusion of a sham control in subsequent studies would strengthen the specificity of tDCS effects over placebo effects, thus enriching the validity of the conclusions.

Taken together, the present study showed remarkable interactions between tDCS and individual factors associated to AD, mainly affecting intra- and inter-network FC, mainly related to SNs. Considering the complex pathogenesis of AD and the variable response to interventions in MCI patients, our findings support precision-targeted treatment strategies adapted to genetic profiles and gender-specific responses. Future investigations should extend the study duration and include a control group to further validate the therapeutic potential of tDCS in the early stages of AD, where treatment options are currently limited.

## Data availability statement

The raw data supporting the conclusions of this article will be made available by the authors, without undue reservation.

## Ethics statement

The studies involving humans were approved by Institutional Review Board of the Catholic University of Korea. The studies were conducted in accordance with the local legislation and institutional requirements. The participants provided their written informed consent to participate in this study.

## Author contributions

DK: Conceptualization, Data curation, Formal analysis, Funding acquisition, Methodology, Project administration, Visualization, Writing – original draft, Writing – review & editing. S-MW: Data curation, Methodology, Writing – review & editing. YU: Investigation, Software, Writing – review & editing. SK: Data curation, Methodology, Writing – review & editing. TK: Data curation, Methodology, Writing – review & editing. DK: Data curation, Methodology, Writing – review & editing. CL: Conceptualization, Supervision, Writing – review & editing. HL: Conceptualization, Methodology, Project administration, Supervision, Writing – review & editing.
