# Supplementary material for: Transcranial direct current stimulation and neuronal functional connectivity in MCI: role of individual factors associated to AD
Source: Front Psychiatry. 2024 Aug 19;15:1428535. doi: 10.3389/fpsyt.2024.1428535 (PMC11366601; doi:10.3389/fpsyt.2024.1428535)
Supplement: Supplementary file 1 [file Table1.docx]

**Transcranial direct current stimulation and neuronal functional connectivity in MCI: Role of individual factors associated to AD**

Dong Woo Kang^a^, Sheng-Min Wang^b^, Yoo Hyun Um^c^, Sunghwan Kim^b^, TaeYeong Kim^d^, Donghyeon Kim^d^, Chang Uk Lee^a^, and Hyun Kook Lim^b*^

^a^Department of Psychiatry, Seoul St. Mary’s Hospital, College of Medicine, The Catholic University of Korea, Seoul, Republic of Korea

^b^Department of Psychiatry, Yeouido St. Mary’s Hospital, College of Medicine, The Catholic University of Korea, Seoul, Republic of Korea

^c^Department of Psychiatry, St. Vincent’s Hospital, College of Medicine, The Catholic University of Korea, Seoul, Republic of Korea

^d^Research Institute, NEUROPHET Inc.; Seoul 06247, Republic of Korea

*Address correspondence to Hyun Kook Lim, MD, PhD

Full address: Department of Psychiatry, Yeouido St. Mary’s Hospital, College of Medicine, The Catholic University of Korea, 10, 63-ro, Yeongdeungpo-gu, Seoul, 06591, Republic of Korea; ﻿Tel.: +82 2 3779 1048; Fax: +82 2 780 6577; E-mail: [drblues@catholic.ac.kr](mailto:drblues@catholic.ac.kr)

# Supplementary Methods

## Neuropsychological evaluation

# Cognitive status was assessed using neuropsychological testing at Yeouido St. Mary’s Hospital, The Catholic University of Korea. Cognitive functions in all the subjects were assessed using the Korean version of the Consortium to Establish a Registry for Alzheimer’s Disease (CERAD-K)^1^, which included verbal fluency (VF), the 15-item Boston Naming Test (BNT), MMSE-K, Word List Memory (WLM), Word List Recall (WLR), Word List Recognition (WLRc), Constructional Praxis (CP), and Constructional Recall (CR). The CERAD is a standardized clinical and neuropsychological assessment battery for the evaluation of patients with AD (1). The results were reviewed by a neuropsychologist to determine whether there was evidence of cognitive impairment.

# The VF score is the number of animal names that the subject could name in one minute. The BNT scores ranged from 0 to 15 points. The MMSE-K score ranged from 0 to 30 points. The WLM scores ranged from 0 to 30 points. The WLR scores ranged from 0 to 10 points. The WLR scores ranged from 0 to 10 points. The WLRc scores ranged from 0 to 10 points. The CP scores ranged from 0 to 11 points. The CR scores ranged from 0 to 11 points. The total memory domain scores ranged from 0 to 67 points. Finally, the total CERAD-K scores ranged from 0 to 100 points. All subdomain scores indicate higher subdomain cognitive function with higher scores.

# To evaluate executive function, we performed the Stroop task, which requires pre-directed reactions while suppressing the dominant response, such as letter reading and color reading conditions. In the current study, the Korean Color Word Stroop Test (K-CWST) was used (2). Participants name the ink color of words where the color name and ink color do not match. The score is the number of correct responses in the given time (3). Additionally, participants carried out trail-making test B (TMTB) (4), which measures the time taken to alternate letters and numbers in a sequence by drawing lines. Finally, the VF test, the subdomain of the CERAD-K battery, counts the number of animals recalled within a minute. Higher scores on the K-CWST and VF indicate higher executive function, while higher scores on the TMTB indicate lower executive function.

## *APOE* genotyping

DNA was isolated from blood using the QIAmp Blood DNA Maxi Kit protocol (Qiagen, Valencia, CA). Genotypes for two APOE SNPs, rs429358 (E*4) and rs7412 (E*2) were determined using TaqMan SNP genotyping assays (Applied Biosystems, Foster City, California).

## BDNF genotyping

For DNA extraction, an aliquot from each saliva sample was added to GeneAll Exgene ™ Clinic SV (Doctor protein INC, Korea) and DNA was extracted according to manufacturer’s instructions. Sample DNA extractions were used in subsequent polymerase chain reaction (PCR) to amplify the target 274-bp BDNF fragment. Following the PCR procedure, the PCR products were run on a 1.5% agarose gel along- side a DNA size ladder at 300 V for 20 min. The agarose gel containing the PCR products was stained with ethidium bromide, visualized under ultraviolet light, and examined for PCR amplification products that represent the amplification of the target 274-bp BDNF fragment. DNA was amplified by using a DNA Engine Tetrad 2 Peltier Thermal Cycler (BIO-RAD, Hercules,CA, USA) and sequenced by using an ABI PRISM 3730XL analyzer (Applied Biosystems, Foster City, CA, USA). Sequencing data were analyzed by using Variant Reporter Software Version 2.1 (Applied Biosystems).

## PET scanners

Each scanner was commissioned by scanning a NEMA phantom and adjusting the reconstruction parameters to obtain a spatial resolution of ~6.5 mm. This optimization was performed before the patients were scanned, and the images received by GE were not subjected to any further post-processing regarding spatial resolution.

# Supplementary References

1. Lee JH, Lee KU, Lee DY, Kim KW, Jhoo JH, Kim JH, et al. Development of the Korean Version of the Consortium to Establish a Registry for Alzheimer's Disease Assessment Packet (CERAD-K) clinical and neuropsychological assessment batteries. *The Journals of Gerontology Series B: Psychological Sciences and Social Sciences* (2002) 57:P47-P53

2. Kim TY, Kim S, Sohn JE, Lee EA, Yoo BG, Lee SC, et al. Development of the Korean Stroop Test and Study of the Validity and the Reliability. *Journal of the Korean Geriatrics Society* (2004) 8:233-40

3. Scarpina F, Tagini S. The Stroop Color and Word Test. *Front Psychol* (2017) 8:557. doi:10.3389/fpsyg.2017.00557

4. Seo EH, Lee DY, Kim KW, Lee JH, Jhoo JH, Youn JC, et al. A normative study of the Trail Making Test in Korean elders. *International Journal of Geriatric Psychiatry: A journal of the psychiatry of late life and allied sciences* (2006) 21:844-52
